# Molecular and phenotypic characterization of *Als1* and *Als2* mutations conferring tolerance to acetolactate synthase herbicides in soybean

**DOI:** 10.1002/ps.3725

**Published:** 2014-03-06

**Authors:** Kay L Walter, Stephen D Strachan, Nancy M Ferry, Henrik H Albert, Linda A Castle, Scott A Sebastian

**Affiliations:** a DuPont PioneerHayward, CA, USA; b DuPont Crop Protection, DuPont Stine-Haskell Research CenterNewark, DE, USA; c DuPont PioneerJohnston, IA, USA

**Keywords:** acetolactate synthase, acetohydroxyacid synthase, sulfonylurea, herbicide resistance, herbicide tolerance, soybean, mutation

## Abstract

**BACKGROUND:**

Sulfonylurea (SU) herbicides are effective because they inhibit acetolactate synthase (ALS), a key enzyme in branched-chain amino acid synthesis required for plant growth. A soybean line known as W4-4 was developed through rounds of seed mutagenesis and was demonstrated to have a high degree of ALS-based resistance to both post-emergence and pre-emergence applications of a variety of SU herbicides. This report describes the molecular and phenotypic characterization of the *Als1* and *Als2* mutations that confer herbicide resistance to SUs and other ALS inhibitors.

**RESULTS:**

The mutations are shown to occur in two different ALS genes that reside on different chromosomes: *Als1* (P178S) on chromosome 4 and *Als2* (W560L) on chromosome 6 (P197S and W574L in *Arabidopsis thaliana*).

**CONCLUSION:**

Although the *Als1* and *Als2* genes are unlinked, the combination of these two mutations is synergistic for improved tolerance of soybeans to ALS-inhibiting herbicides. © 2014 DuPont Pioneer. *Pest Management Science* published by JohnWiley & Sons Ltd on behalf of Society of Chemical Industry.

## INTRODUCTION

The class of chemistry known as sulfonylurea (SU) herbicides was discovered by DuPont in 1975, and the first SU herbicide products were commercialized in 1982. SUs provided farmers with the first high-specificity herbicides that could be used at very low use rates. Thirty years after their introduction, SUs are still sold in over 80 countries for use on over 25 crops.

SUs are effective herbicides because they inhibit acetolactate synthase (ALS), also known as acetohydroxyacid synthase (AHAS), a key enzyme in branched-chain amino acid synthesis required for plant growth.^1^ As this enzyme is absent in animals, SUs have very low toxicity to non-target species. These qualities make SUs valuable tools for controlling weeds in a wide variety of applications. Some SUs are non-selective (effective on all plants) and can be used for complete vegetation control in industrial settings. Others are selective, i.e. they act on some plant species but are tolerated by others, based on the plant's differential ability to detoxify the SU before significant inhibition of ALS activity.

To date, two specific SUs (chlorimuron ethyl and thifensulfuron) are registered and used in a variety of herbicide formulations for selective weed control in soybean. Wild-type soybeans tolerate these SUs through metabolic inactivation.^2^,^3^ However, higher resistance to these and other SUs can also be conferred through specific mutations within ALS gene(s) that make the enzyme less susceptible to SU inhibition while retaining vital catalytic activity.^4^,^5^

In the mid-1980s, mutation-breeding techniques were used to develop a soybean line called ‘W20’ (derived from the cultivar Williams) with ALS-based resistance to SU herbicides.^6^,^7^ W20 is the original source line of the soybean trait known commercially as STS® (sulfonylurea-tolerant soybean). STS® offers more selectivity and flexibility with SUs specifically registered for soybean and may provide options for the use of more efficacious and/or broad-spectrum SUs for weed control.

Seed of W20 was released broadly to all major soybean-breeding companies in both North and South America in the late 1980s. Although breeding and commercial use of the STS® trait expanded quickly in the early 1990s, this expansion slowed in favor of transgenic glyphosate resistance (the Roundup Ready® or RR® trait) by the mid-1990s. This shift was driven mainly by the simplicity and convenience to the applicator to apply only one herbicide for broad-spectrum weed control, the improved control of weeds developing resistance to other herbicidal modes of action and the improved soybean crop tolerance to glyphosate relative to the combinations of several of the commercial herbicides labeled for use in soybean at that time. Although ‘stacked’ (RR® + STS®) varieties have been available since the mid-1990s, there is now renewed interest in trait stacks to provide more options for the control of glyphosate-resistant weeds.^8^,^9^

Inheritance studies^6^ confirmed that whole-plant SU resistance of W20 was conferred by a single semi-dominant mutation, which was later found to be a proline-to-serine substitution at position 178 in a soybean ALS gene (Falco SC, unpublished), equivalent to P197S in *Arabidopsis thaliana*, that cosegregated with *in vitro* resistance of the ALS enzyme to SU inhibition. With this evidence, the name ‘*Als1*’ was given to the new allele conferring SU resistance in W20. As *Als1* provides a high level of SU resistance compared with the wild-type ‘*als1*’ allele, simple and reliable phenotypic screens can distinguish between plants that are wild type (*als1*/*als1*), heterozygous (*Als1*/*als1*) and homozygous (*Als1*/*Als1*) in segregating breeding populations. Therefore, there was initially less incentive to develop genetic markers for marker-assisted selection (MAS) of *Als1*.

Subsequent to the development of W20, lines homozygous for *Als1* were subjected to a second round of mutagenesis in an attempt to derive mutations that confer even higher levels of SU resistance than that provided by *Als1* alone. From the second round of mutagenesis, a line ‘W4-4’ was selected and proven to be more resistant to SUs than the original W20 line via both *in vitro* ALS enzyme and whole-plant assays.^7^

Segregation for SU resistance within populations derived from W4-4 × wild-type crosses indicated that W4-4 was homozygous for *Als1* plus a second independently segregating mutation herein called ‘*Als2*’ (Sebastian SA, unpublished). Other studies demonstrated repeatedly that the combination of *Als1* and *Als2* provides higher crop safety for virtually every SU tested, including broad-spectrum SUs that could be registered for use in soybean lines containing both *Als1* and *Als2*.

Given the increasing incidence of glyphosate-resistant weeds, there is renewed interest in both *Als1* and *Als2* to expand future weed control options in soybean. To this end, incorporation of both mutations into elite soybean germplasm via conventional breeding methods is under way. Breeding efforts to stack *Als1* and *Als2* along with other desirable traits (both native and transgenic) could be greatly facilitated by the development of codominant SNP (single nucleotide polymorphism) markers for MAS. This would eliminate the need for phenotypic screening protocols that can differentiate the numerous zygosity states possible in breeding populations. Knowing the exact zygotic condition of single plants would reduce the need for progeny testing to confirm that breeding lines are ‘fixed’ (homozygous and homogeneous) for both *Als1* and *Als2* alleles. Efforts to backcross both genes into the latest elite germplasm would also be simplified with MAS. Hence, the following studies were conducted to determine the DNA sequence of the *Als1* and *Als2* alleles so that broadly applicable SNP markers could be developed for MAS of these valuable alleles. As W4-4 was known to contain both *Als1* and *Als2* mutations, based on its breeding history and SU-resistant phenotype, sequencing of these alleles was accomplished by comparing the sequence of ALS genes in W4-4 with the sequence of ALS genes in wild-type soybean lines.

## MATERIALS AND METHODS

### Sequencing studies

#### Genomic DNA extraction

Genomic DNA from W4-4 seeds was extracted at room temperature. Root sections, approximately 0.5 cm long, were removed from two germinated seeds on the fourth day and added to a 2 mL microfuge tube along with two stainless steel 5/32″ balls. The tubes were placed in a GenoGrinder 2000 to grind the tissue with settings 1 × 250 strokes min^−1^ for 1 min and 30 s. The tube was then microcentrifuged at 13 000 × *g* for 1 min. The root samples were ground and microcentrifuged a second time at the same settings, and genomic DNA was then isolated from the supernatant using the Qiagen DNeasy Plant Mini kit (Cat. No. 69104) as per the manufacturer's protocol.

#### Molecular cloning of the genomic DNA for the catalytic subunit of the ALS gene family

The cDNA sequence for the *Als1* catalytic subunit was used to search a proprietary soy sequence database with the blastn program.^10^ Nine sequences described as acetolactate synthase were identified and mapped to the JGI/DOE soy genomic sequence assembly Glyma1^11^ for wild-type genotype Williams 82. These nine sequences corresponded to five loci annotated as ALS genes listed in Table1. The gene on chromosome 1 with introns excised has an open reading frame length of 278 amino acids, which is less than half the size of the next smallest ALS gene on chromosome 4, which has 641 residues. It was unclear whether the chromosome 1 gene was a functional ALS gene or a pseudogene. Nevertheless, this gene was included in the sequencing analysis. PCR primers were designed to flank the genes predicted by the Fgenesh algorithm at these loci (Table2). Using W4-4 genomic DNA as the template, PCR products for the ALS genes were cloned using Finnzymes Phusion DNA polymerase (Cat. No. F-530S), following the instructions for recommended temperatures and cycles in the Phusion protocol. The PCR products were cloned into the vector pCR-BluntII-TOPO and transformed into TOP10 cells (Invitrogen Cat. No. K2800-20). Sequencing was done by Sequetech (Mountain View, CA). Sequences were aligned with Sequencher 4.8 software.

**Table 1:** Gene names for ALS catalytic subunit genes from the soybean genome

Gene name	Fgenesh	Complete ORF length (AA)	Wild-type allele name
Glyma01g09920.1	GM01_5367	278	
Glyma04g37270.1	GM04_21145	641	*als1*
Glyma06g17790.1	GM06_4197	645	*als2*
Glyma13g31470.1	GM13_13846	645	
Glyma15g07860.1	GM15_1662	653	

**Table 2:** Oligonucleotide primers used for cloning of ALS genes from W4-4 soybean genomic DNA

Primer name	Primer sequence (5′ to 3′)
GM01F	GAAACTCTCCACCGCCTC
GM01R	GATCACTAAGTAACCATTAAAGAC
GM04F	TTAATAAATTTTCTACATCCCAGTGA
GM04R	GATGCTACTGCATGTAGTAAG
GM06F	GACACACTCTGAGAGTCTC
GM06R	TACCAAAACTACTGCAAACTATG
GM13F	ACCTAAGTTAATTCATGAAATGTTTG
GM13R	GCTATATTAGCTTACTATTTTTACAAAAC
GM15F	GATCATTAAACGTTTTAACGCG
GM15R	TATCTTAGTTGCCAACATGAATAC

#### *Cloning of* Als1 *and* Als2 *cDNAs from W44*

Two 6 mm leaf punches per tube were taken from the first trifoliate leaves of four-week-old W4-4 plants and stored at −80 °C. Total RNA from the plant tissue was purified using the Qiagen RNeasy Mini Plant kit (Cat. No. 74904), with the frozen tissue ground in a GenoGrinder 2000 set to 1 × 250 strokes min^−1^ for 1 min after the addition of two steel 3/32″ balls and kit lysis buffer containing beta mercaptoethanol in each tube. Synthesis of cDNA was done with Invitrogen's 3′ RACE kit (Cat. No. 18373019) using 1 µg of total RNA as template following the manufacturer's protocol. A no-reverse-transcriptase control was also included. Finnzymes Phusion DNA polymerase (Cat. No. F-530S) was used for PCR, with the cDNA as starting template, following the instructions for recommended temperatures and cycles in the Phusion protocol. Forward primers were designed in the 5′ untranslated regions for the two genes that were found to contain mutations located on chromosomes 4 and 6. Reverse primers were located in the gene near the stop codon and in the terminator region. The PCR primers are listed in Table3.

**Table 3:** Oligonucleotide primers used for cloning of *Als1* and *Als2* genes from W4-4 soybean cDNA

Primer name	Primer sequence (5′ to 3′)
ALS1F	TGGTGCTACCCACACAACAC
ALS2F	CAGTGCAGCCACACAAAGAC
ALS3′UTRR	CTCACCACAGGCCAAATC
ALSR	CATCCTTGAAGGATCCATTACTGGGAATCA

#### Reverse transcription quantitative PCR

To confirm the *Als1* and *Als2* expression results from cDNA cloning, reverse transcription quantitative PCR (RT-qPCR) was performed. Approximately 6 µg of total RNA that was isolated as explained in the previous section was DNase treated with the Qiagen RNase-Free DNase set (Cat. No. 79254) by following the manufacturer's RNA Cleanup protocol with DNase on-column digestion in the Qiagen RNeasy Mini kit (Cat. No. 74104). Synthesis of cDNA was performed using Invitrogen's 3′ RACE kit (Cat. No. 18373019) with 870 ng of total RNA as template. A no-reverse-transcriptase control was also included. The cDNA and the no-reverse-transcriptase control were both diluted with 60 µL of Tris-EDTA (pH 8.0) buffer. Forward and reverse primers were used to amplify regions of approximately 200 bp length for *Als1* and *Als2*. Primers to amplify a region in the *Glycine max* eukaryotic initiation factor 4A-15-like (eIF-4A) gene (RefSeq accession NM_001255135.2) were used as a control. All primer sequences used for RT-qPCR are listed in Table4. PerfeCTa SYBR Green Supermix UNG (Quanta Biosciences Cat. No. 95068–500) was used for each reaction, which was scaled down to 25 µL from the manufacturer's original 50 µL protocol, as well as 2 µL of diluted cDNA (or no-RT control) and 1.25 µL of each 10 µM primer. Each template/primer pair reaction was done in triplicate. Amplification was performed with a Bio-Rad Chromo4 real-time detector with a DNA engine thermal cycler. Initial incubation was at 50 °C for 2 min, followed by denaturation at 95 °C for 2 min. Forty PCR cycles were performed: denaturation at 95 °C for 15 s, annealing at 58 °C for 10 s and elongation at 72 °C for 15 s. This was followed by a melting curve program reading every 0.2 °C from 65 to 85 °C. Opticon Monitor 3.1.32 software was used to analyze the results.

**Table 4:** Oligonucleotide primers used for reverse transcription quantitative PCR (RT-qPCR)

Primer name	Primer sequence (5′ to 3′)
ALS1QF	CTTCACCAAGGAAGCGC
ALS1QR	TTCGGCGGCGAAGAC
ALS2QF	CGCCGGCAACATCAG
ALS2QR	TCGGCGGCGAAGATG
EIF4AQF	ATGCTGCGCAGACAGTCACT
EIF4AQR	CAGCCTCATCCAATACAAACATCT

### Phenotypic analyses

#### Evaluation of herbicide tolerance

Soybean isolines having wild-type *als1* and *als2* genes, the *Als1* mutation, the *Als2* mutation and both the *Als1* and *Als2* mutations were evaluated for their responses to eleven herbicides representing the five chemical families that inhibit the ALS enzyme. The five chemical families represented were: (1) the imidazolinones; (2) the pyrimidinylthiobenzoates; (3) the sulfo-nylaminocarbonyltriazolinones (also known as the triazolinones); (4) the sulfonylureas; (5) the triazolopyrimidines. Soybean seeds from each line were planted into 10 cm pots filled with Redi-Earth potting mix (Sun Gro Horticulture Canada Ltd, Alberta), and the resulting plants were grown in a greenhouse environment supplemented with lighting (16 h photoperiod) for the duration of the test. Herbicide active ingredients were applied as formulated materials in water containing 0.25% (v/v) non-ionic surfactant post-emergence to soybeans at the V2 growth stage.^12^ Herbicides were applied as post-emergence broadcast treatments in a spray volume of 280 L ha^−1^. Application rates tested were 0.5×, 1×, 2×, 4× and 8× the label rate for each active ingredient. Label rates (g AI = active ingredient ha^−1^) for each active ingredient were: imazapyr 17.5; imazethapyr 70; pyrithiobac sodium 70; chlorimuron 17.5; nicosulfuron 35; rimsulfuron 17.5; sulfometuron 8.75; thifensulfuron 8.75; tribenuron 8.75; flucarbazone 35; cloransulam methyl 17.5.

Phenotypic plant response was visually evaluated and recorded 14 days after herbicide application. Response scores were based on a 0–100 scale, where zero is no visual response and 100 is plant death. Controls consisted of soybean plants of the same variety that received no herbicide treatments for each of the varieties tested. There were two replications of each soybean line for each herbicide treatment.

Log-logistic dose–response curves were fitted to percentage phenotypic responses using log_10_ of dose.^13^ Curves were fitted using the logit link with normal errors. Inverse prediction was used to calculate the dose of herbicide estimated to elicit 50% (EC_50_) or 10% (EC_10_) response. The 95% confidence intervals for EC*_x_* values were calculated using Fieller's theorem.^14^ These EC*_x_* values and their confidence intervals were inputs to test for the interaction between the *Als1* and *Als2* genes using the isobole method of analysis. If phenotypic plant response to the highest dose of the herbicide was at least 80% for the *Als1*, *Als2* and *Als1* + *Als2* biotypes, EC_50_ values were used to test for synergistic interactions between the *Als1* and *Als2* genes for improved tolerance to the herbicide. If phenotypic plant response to the highest dose of the herbicide was 20% or less for any one of the three biotypes, EC_10_ values were used to test for synergism.

#### Isobole method of analysis

Isoboles are a commonly used approach for assessing possible interaction. The historic basis for predicting the effect of a combination is based on the concept of dose equivalence, i.e. an equally effective dose (a) of one will add to the dose (b) of the other in a combination situation.^15^ Isobole analysis reduces phenotypic plant response plotted in three-dimensional space (*X* = contribution of *Als1* gene, *Y* = contribution of *Als2* gene, *Z* = plant response to the herbicide) to a two-dimensional plot by selecting a quantitatively defined effect as criterion for a graphic representation of the joint action of the two components (e.g. *X* = dose of herbicide estimated to produce 50% phytotoxicity when the *Als1* gene is present and *Y* = dose of herbicide estimated to produce 50% phytotoxicity when the *Als2* gene is present).^16^,^17^ In his research paper, Tammes^18^ quotes Loewe and Muischnek's studies^19^ on the joint action of drugs, naming such a line an ‘isobole’, a line of equal effect (zero interaction). The isobole method is generally valid, regardless of mechanisms of action.^20^

## RESULTS

### Sequencing results/alignments

**Figure 1:**
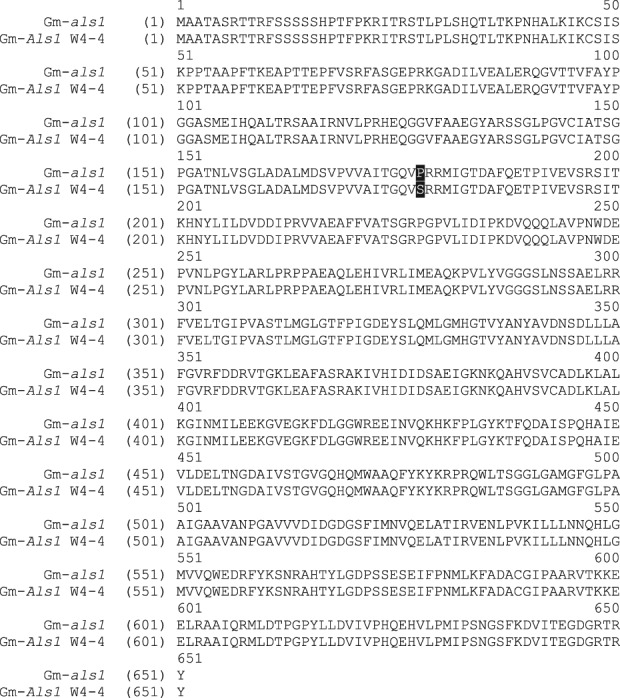
Alignment of wild-type *als1* with W4-4 *Als1* with mutation P178S.

**Figure 2:**
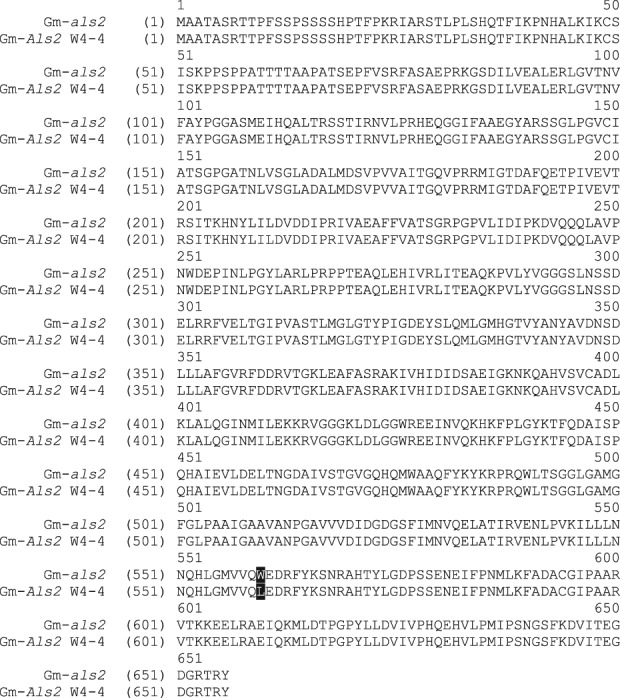
Alignment of wild-type *als2* with W4-4 *Als2* with mutation W560L.

Sequencing results for the five ALS genes showed a mutation for two out of the five genes in Table1, Glyma04g37270.1 and Glyma06g17790.1. The mutant allele in Glyma04g37270.1 is *Als1* and the mutant allele in Glyma06g17790.1 is proposed as *Als2*. Alignments of the full-length wild-type *als1* and *als2* alleles with their *Als1* and *Als2* counterparts were made with the AlignX feature in Vector NTI software (Invitrogen). Results are shown in Figs. 1 and 2. W4-4's *Als1* allele resulted in a proline-to-serine substitution at position 178 of the full-length soybean protein (P197S in *A. thaliana*), which confirms the mutation previously found in W20 (Falco SC, unpublished). W4-4's *Als2* allele resulted in a tryptophan-to-leucine substitution at position 560 of the full-length soybean protein (W574L in *A. thaliana*). These mutations are similar to the mutations in the highly herbicide-resistant ALS variant known as HRA,^21^–^23^ which, in soybean, has mutations P178A and W555L in the same ALS gene (P197A and W574L in *A. thaliana*). Amino acid position 555 in *Als1* is analogous to amino acid 560 in *Als2*.

### Expression of *Als1* and *Als2* mutations

**Figure 3:**
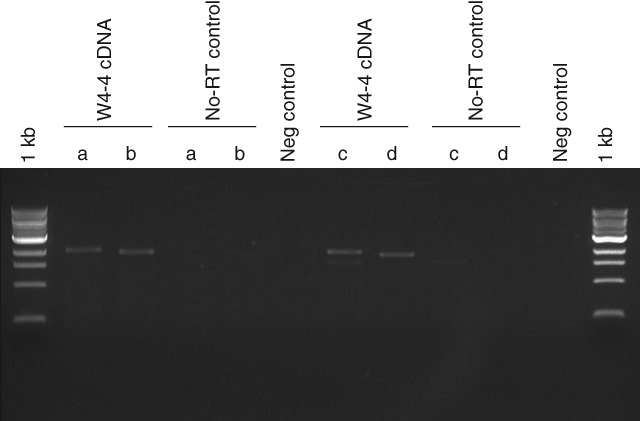
*Als1* and *Als2* alleles were amplified from W4-4 cDNA with primers from Table3: a – ALS1F and ALS3′UTRR; b – ALS1F and ALSR; c – ALS2F and ALS3'UTRR; d – ALS2F and ALSR. Faint bands in no-RT control lanes are slight genomic contamination in the RNA preparation used for cDNA synthesis.

**Figure 4:**
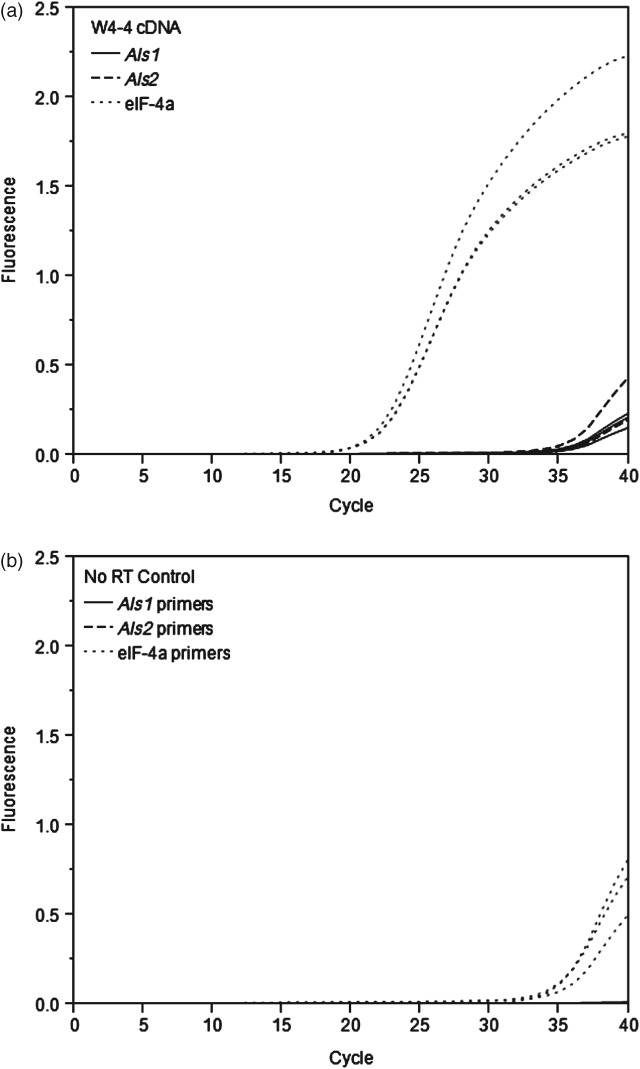
Reverse transcriptase quantitative PCR results for *Als1*, *Als2* and eIF-4A genes: (a) with W4-4 cDNA only; (b) with no-RT control only (all done in triplicate).

Results of the cDNA cloning of the *Als1* and *Als2* mutations are shown in the 1% Tris-borate-EDTA (TBE) agarose gel in Fig.3 [30 min at 100 V, 1 kb ladder (NEB Cat. No. N0468S)]. The amplified cDNA fragments appear to match the expected fragment sizes of 2066 bp and 1942 bp for *Als1* (see Fig.3, lanes marked ‘a’ and ‘b’ respectively) and 2083 bp and 1957 bp for *Als2* (see Fig.3, lanes marked ‘c’ and ‘d’ respectively). The faint bands in the no-RT control lanes can be attributed to slight genomic contamination, as the original RNA isolation preparation was not DNase treated. The remaining RNA prep was subsequently DNase treated before cDNA synthesis and RT-qPCR. The RT-qPCR results are shown in quantification cycle (Cq) plots in Fig.4, where Cq is the terminology adopted in the MIQE guidelines.^24^ Lower Cq values indicate higher levels of the specific mRNA being analyzed. The Cq values are also listed in Table5. The Cq values for *Als1* and *Als2* were higher than the Cq value for the eIF-4A control in the W4-4 sample. This suggests that the expression of *Als1* and *Als2* was relatively low compared with eIF-4A. The no-RT control did not register Cq values for *Als1* and *Als2*. Although the eIF-4A/no-RT combination had an average Cq value of 34.93, this value is significantly higher than the average Cq value of 21.41 for eIF-4A in the W4-4 sample, and the genomic contamination can be regarded as insignificant. The ratio of *Als2* to *Als1* is calculated using the formula^25^

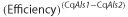


**Table 5:** Cq values for RT-qPCR results

Sample	Gene	Efficiency	Cq	Average Cq	Maximum Cq	Minimum Cq	Cq SD
W4-4 cDNA	*Als1*	1.62	37.56	37.64	38.23	37.21	0.43
W4-4 cDNA	*Als2*	1.68	37.04	37.14	37.7	36.18	0.68
W4-4 cDNA	*eIF-4a*	1.78	21.39	21.41	21.52	21.21	0.14
No-RT control	*Als1*	N/A	N/A	N/A	N/A	N/A	N/A
No-RT control	*Als2*	N/A	N/A	N/A	N/A	N/A	N/A
No-RT control	*eIF-4a*	1.67	34.89	34.93	35.56	34.52	0.45

where the average of the efficiencies for *Als1* and *Als2* was used, as well as the averages of the Cq values. This resulted in a ratio of 1.30, which suggests that the level of expression of *Als1* and *Als2* is essentially the same.

Together, the cDNA cloning and the RT-qPCR results confirm the expression of both of the *Als1* (P178S) and *Als2* (W560L) genes, suggesting that the mutations in both of these genes contribute to the high levels of sulfonylurea tolerance in the W4-4 soy line.

### Evaluation of herbicide tolerance

**Figure 5:**
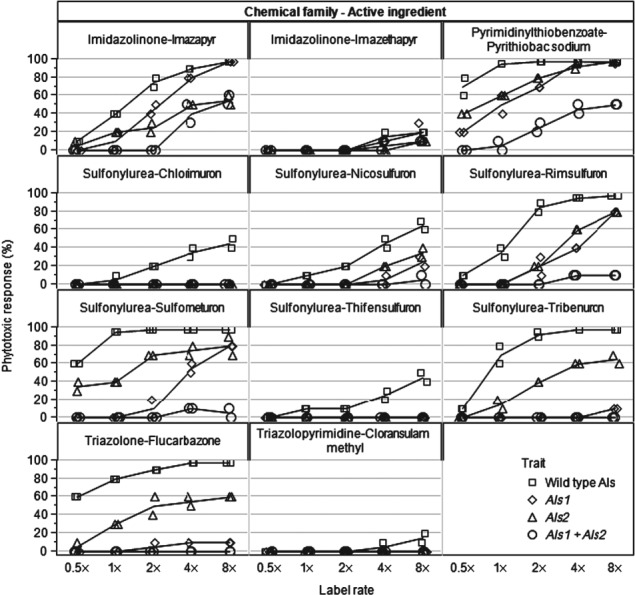
Soybean response to post-emergence applications of ALS herbicides.

*Als1*, *Als2* and the *Als1* + *Als2* combination dramatically reduced soybean response to post-emergence applications of herbicides inhibiting the acetolactate synthase enzyme (Fig.5). Based on visual analysis of dose–response curves, when compared with the wild type, *Als1* significantly improved soybean tolerance to chlorimuron, nicosulfuron, rimsulfuron, sulfometuron, thifensulfuron, tribenuron and flucarbazone. When compared with the wild type, *Als2* improved soybean tolerance to imazapyr, chlorimuron, nicosulfuron, rimsulfuron, sulfometuron, thifensulfuron, tribenuron and flucarbazone. When compared with the wild type, inclusion of the combination of *Als1* + *Als2* improved soybean tolerance to imazapyr, pyrithiobac sodium, chlorimuron, nicosulfuron, rimsulfuron, sulfometuron, thifensulfuron, tribenuron and flucarbazone. When compared with soybean containing *Als1* only or *Als2* only, inclusion of the combination of *Als1* + *Als2* improved soybean tolerance to imazapyr, pyrithiobac sodium, nicosulfuron, rimsulfuron, sulfometuron and flucarbazone. These test results confirm that the inclusion of *Als1*, *Als2* and the combination of *Als1* + *Als2* improve the tolerance of soybean to at least four of the five chemical families active on ALS. Wild-type soybean showed little phytotoxic response to imazethapyr or cloransulam methyl. Both of these herbicides are inherently selective for soybean tolerance and are labeled for weed control in soybean. Test results for imazethapyr and cloransulam methyl show that *Als1*, *Als2* and the combination of *Als1* + *Als2* do not increase the sensitivity of soybean to either herbicide.

**Figure 6:**
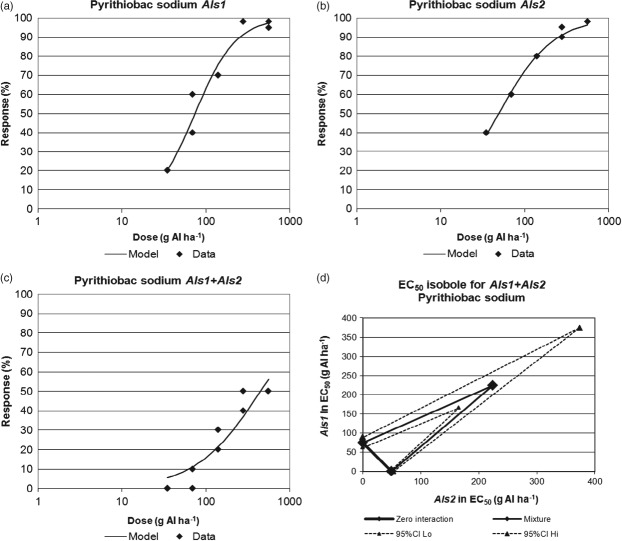
Soybean response to post-emergence applications of pyrithiobac sodium: (a) *Als1* only; (b) *Als2* only; (c) *Als1* + *Als2*; (d) isobole plot based on EC_50_ for *Als1* + *Als2*, illustrating synergism versus the zero interaction line.

Data presented in Fig.5 were used to estimate doses of herbicides that elicit either 10% or 50% phytotoxicity response of ‘wild-type’ soybean and soybean containing the *Als1*, *Als2* or *Als1* + *Als2* genes. These EC_10_ and EC_50_ values, and their confidence intervals, were subsequently used for isobole analysis to test for zero interaction, antagonism or synergism among the various ALS genotypes. For example, in Fig.6, the predicted dose–response curves based on log-logistic analysis are compared with the observed data for pyrithiobac sodium for the three soybean biotypes. Amounts of pyrithiobac sodium to cause 50% phytotoxicity were estimated to be 74 (62–89) g AI ha^−1^, 49 (46–53) g AI ha^−1^ and 450 (330 to >560) g AI ha^−1^ [mean (95% confidence interval)] for soybean containing *Als1* only, *Als2* only or *Als1* + *Als2* genes respectively. These EC_50_ values were then compared to test for interactions between the *Als1* and *Als2* genes.

The isobole method of analysis for gene interaction is based on the dose response of biologically active agents in combination and uses ‘isoeffective’ or equally effective doses for each of the components to build an isobole graph. In this study, the agents being evaluated were the *Als1* and *Als2* gene mutations. Soybean responses to increasing doses of herbicides were used to assess the effectiveness of each gene to improve tolerance to ALS-inhibiting herbicides. If there is no interaction between the genes, an isoeffective amount of one gene can be substituted for the other gene. The zero interaction line in the isobole is a straight line connecting isoeffective rates (e.g. EC_50_) for each of the two genes (Fig.6d). An EC_50_ line with confidence intervals was estimated for the observed response of soybean containing the combination of *Als1* + *Als2*. These values were then plotted on the isobole graph by parsing out the contribution to the (*Als1* + *Als2*) combination's EC_50_ from its *Als1* and *Als2* components using a 1:1 ratio assumption. A 1:1 ratio is assumed because *Als1* + *Als2* consists of a single mutation of *Als1* on one ALS gene and a single mutation of *Als2* on an independent ALS gene. If the zero interaction line is below the lower confidence interval for the combination, synergy is indicated. In addition, if the combination of the *Als1* and *Als2* genes is synergistic in their ability to improve soybean tolerance to ALS herbicides, substantially more herbicide is required to produce the same level of phytotoxic response in soybean containing both ALS genes than in soybean containing only one of the ALS genes. For example, substantially more pyrithiobac sodium (450 g AI ha^−1^) was required to cause 50% phytotoxicity in the *Als1* + *Als2* biotype versus the amount of pyrithiobac sodium necessary to cause a 50% phytotoxic response in either the *Als1* only (74 g AI ha^−1^) or the *Als2* only (49 g AI ha^−1^) biotypes (Fig.6). Synergistic activity of the combination of *Als1* plus *Als2* genes to improve soybean tolerance to ALS herbicides was observed for imazapyr, pyrithiobac sodium, rimsulfuron, sulfometuron, tribenuron and flucarbazone (Table6). These active ingredients represent four of the five chemical families with herbicidal activity on the acetolactate synthase enzyme, suggesting that the combination of *Als1* + *Als2* provides improved tolerance of soybean to all chemistries with activity on this site of action. Isobole analysis was not conducted for imazethapyr, chlorimuron, thifensulfuron or cloransulam methyl because insufficient herbicide was applied to produce substantial phytotoxicity in soybean containing either the *Als1* or the *Als2* gene and in soybean containing both genes. Wild-type soybean can tolerate these specific herbicides through independent (non-ALS-based) metabolic mechanisms. Although there was insufficient soybean response to test for synergism for imazethapyr, chlorimuron, thifensulfuron or cloransulam methyl, data from Fig.5 show that inclusion of the *Als1*, *Als2* and *Als1* + *Als2* mutations at least maintains and can dramatically improve soybean tolerance to these herbicides.

**Table 6:** Dose response and isobole analysis data of soybeans treated with ALS herbicides

Chemical family	Active ingredient	EC isobole	Biotype^a^				Geneinteraction
			Wild type (*als1* + *als2*)	*Als1 only*	*Als2 only*	*Als1* + *Als2*	
			(g AI ha^−1^)	(g AI ha^−1^)	(g AI ha^−1^)	(g AI ha^−1^)	
Imidazolinone	Imazapyr	EC_50_	22 (20–23)	39 (34–44)	94 (71–140)	110 (87 to >140)^b^	Synergistic
Pyrimidinylthiobenzoate	Pyrithiobac sodium	EC_50_	<35^c^	74 (62–89)	49 (46–53)	450 (330 to >560)	Synergistic
Sulfonylurea	Nicosulfuron	EC_10_	38 (26–49)	190 (90–220)	110 (60–140)	>280	Zero interaction
Sulfonylurea	Rimsulfuron	EC_10_	10 (8–12)	29 (18–37)	24 (18–29)	120 (80 to >140)	Synergistic
Sulfonylurea	Sulfometuron	EC_10_	<4.4	15 (9–19)	<4.4	>70 (17 to >70)	Synergistic
Sulfonylurea	Tribenuron	EC_10_	4 (<4–5)	70 (70)	5 (<4–8)	>70	Synergistic
Triazolinone	Flucarbazone	EC_10_	<17.5	230 (120 to >280)	<17.5	>280	Synergistic

a Values are means (95% confidence intervals).

b Values with > are higher than the highest dose of herbicide tested.

c Values with < are lower than the lowest dose of herbicide tested.

ALS genes in other crops, including maize (*Zea mays* L.), wheat (*Triticum aestivum* L.), oilseed rape (*Brassica napus* L.), rice (*Oryza sativa* L.) and sunflower (*Helianthus annuus* L.), have been modified to improve the tolerance of these crops primarily to the imidazolinone herbicides, one of the five chemical families with activity on ALS.^26^ Tolerance to imidazolinone herbicides improved dramatically in maize, oilseed rape, rice and wheat when serine was replaced with asparagine at position 653 (positions in this section reference *A. thaliana*). In maize, a mutation from tryptophan to leucine at position 574 improved maize crop tolerance to the imidazolinone, sulfonylurea, triazolopyrimidine and pyrimidinylthiobenzoate chemical families. Consistent with these results in maize, in the present studies, a mutation from tryptophan to leucine at position 574 improved soybean tolerance to all five chemical families with ALS activity. For the imidazolinone-tolerant crops, oilseed rape and spring wheat, two mutations of ALS loci were required to produce crop hybrids or varieties with sufficient tolerance to be treated commercially with an imidazolinone herbicide for weed control.^26^ In oilseed rape, the two loci were unlinked and additive for improved tolerance to the herbicide. No information is given regarding how the two loci in spring wheat interact. In these studies with soybean, mutations at the two independent loci (positions 197 and 574) acted synergistically for improved crop tolerance to several of the ALS herbicides from different chemical families.

## DISCUSSION

The genetic sequence information from this study has confirmed that the *Als1* and *Als2* mutations are indeed caused by base substitutions within the coding regions of known ALS genes. This study has also confirmed previous evidence that *Als1* and *Als2* are unlinked and on different chromosomes (GM04 and GM06 respectively). Although the *Als1* and *Als2* mutations are not linked, the combination of these two mutations is synergistic for improved tolerance of soybean to ALS-inhibiting herbicides. Most importantly, knowledge of the exact DNA sequence changes can be used to develop genetic markers that are optimized for detection of the causative SNPs responsible for the SU-resistant phenotypes. Markers designed around causative SNPs are preferred to markers that are merely linked with the trait of interest. Even closely linked markers could be linked in repulsion in some germplasm and/or decoupled from the causative SNP by recombination in future breeding cycles. Optimized markers will facilitate rapid and precise incorporation of these commercially useful mutations into a wide variety of elite soybean germplasm.
